# 

**Published:** 1897-02

**Authors:** 


MARRIAGE.

On November 19th, at the residence of the bride's parents, Macedonia, Bradford County, Pa., by the Rev. Mr. Fairchild, Dr. Clarence J. Marshall, of Philadelphia, to Miss Augusta Stevens.

Secretary.

### WISCONSIN SOCIETY OF VETERINARY GRADUATES.

THE annual meeting of this Society will be held at Madison, 1.30 P.M., February 5, 1897. Papers will be presented on "Laminitis," by Dr. B. L. Clark, Monticello; "Tuberculosis," Dr. J. J. Oberst, Cedarburg; "Reflex Irritation," Dr. H. A. Arpke, Sheboygan. Reports of cases b y Drs. Newton, Laws, and Roberts.

W. G. CLARK,
Secretary, Beaver Dam, Wisconsin.

### PENNSYLVANIA STATE VETERINARY MEDICAL ASSOCIATION.

THE annual meeting of this Association will convene at Philadelphia on March 2 and 3, 1897. Papers by Dr. Charles Williams on "Cannabis Indica;" Dr. Leonard Pearson, "Tetanus;" Dr. M. E. Conard, "Abortion;" Dr. John R. Hart, "Azoturea;" Dr. F. F. Hoffman, "Eversion of the Uterus and Vagina." Dr. John Adams and Dr. W. L. Rhoads will also present papers. The subject of tuberculosis will be a topic for consideration.

### PERSONAL.

Veterinarian E. M. Gatchell, of West Chester, Pa., has been named as a candidate for the office of postmaster of that town.

At the last meeting of the New York County Veterinary Medical Association a meeting of the officers for the ensuing year was arranged for in advance of the meeting. Dinner was served, during which time the business and year's work were discussed. It proved a pleasant innovation, and starts the Society with definite lines of work.

Dr. J. H. Wattles, of Kansas City, Mo., is preparing himself for the practice of human medicine, upon which he expects to enter the coming summer.

Dr. J. E. Craig, of Potsdam, New York, has met with the misfortune of having all his books, papers, etc., burned.

Arrangements have been completed by the horseshoers of Greater New York to have a series of lectures given on the anatomy and physiology of the foot by Prof. H. D. Gill at the New York College of Veterinary Surgeons during the next two months.

Dr. Harry D. Paxson, of Wilmington, Del., has received an appointment as meat-inspector under the Bureau of Animal Industry, and been assigned to duty at Chicago, Ill.

Veterinarian Sol. K. Hoffman, of Hamburg, Pa., is a member of the local judiciary, and dispenses justice in his bailiwick.

Veterinarian W. L. Zuill, of Philadelphia, has entered the field of practice of human medicine.

Dr. S. G. Burkholder, of Denver, Pa., has received an appointment under the Bureau of Animal Industry, and been assigned to duty at Chicago, Ill.

Dr. Webb, the noted breeder of hackneys in Vermont, is not relinquishing the breeding of these horses, as announced, but will reduce the number of his stock, so that more time may be spent in developing the others.

State Veterinarian Leonard Pearson, of Pennsylvania, was a visitor to the Bay State during the holiday season.

Veterinarian A. B. Campbell, of Berlin, Ontario, has received an appointment by the Dominion Government as district stock-inspector, under the regulations now existing between the United States and Canada bearing upon import regulations.

Dr. Cooper Curtice, of Moravia, N. Y., having finished the special work for which he was selected by the Bureau of Animal Industry, announces in a card in several of the agricultural stock journals his desire to render services as consulting veterinarian in infectious and contagious diseases of live-stock.

Dr. W. L. Williams, of Ithaca, N. Y., spent a portion of the holiday season on a trip to the West.

Dr. L. O. Lusson, of Ardmore, Pa., has declined the assignment of the Bureau of Animal Industry to Buffalo, N. Y.

Profs. James L. Robertson, H. D. Gill, of New York, and W. Horace Hoskins, of Philadelphia, were interested visitors to the National Poultry and Pigeon Show at Madison Square Garden during the holidays.

Drs. F. G. Scannell, of New York, and E. C. Switzer of Massachusetts, students of the United States Colleges of Veterinary Surgeons of Washington, D. C., have severed their connection therewith, and returned to their homes.

The Board of Trustees of the University of Pennsylvania have elected Dr. John W. Adams to the full professorship of Veterinary Surgery and Obstetrics in the Veterinary Department of that institution.

President Osgood has been favored by the acceptance of Prof. Theobald Smith, of the Chairmanship of the Committee on Diseases for the ensuing year in the U. S. V. M. A.

Veterinarian Simpson, of Northwest Territory, reports increased interest among the stock-breeders in spaying, and larger numbers are being operated upon each year.

The firm of Messrs. Carter & Waugh, of Pittsburg, Pa., has dissolved. The hospital practice will be continued by Dr. Waugh. Dr. Carter will embark in the preparation of veterinary proprietary remedies.

Veterinarian Hugh Le Jambre, of Bordentown, N. J., was seriously injured by his horse running away ; his carriage was wrecked and horse injured also.

Veterinarian Charles Dohan was a keen follower of the hounds of the Lima Hunt Club in its great hunt in January.

In January Dr. R. G. Webster, of Salem, N. J., lost his eldest child by diphtheria, with the disease still afflicting two other of his children.

